# The chromatin-associated RNAs in gene regulation and cancer

**DOI:** 10.1186/s12943-023-01724-y

**Published:** 2023-02-07

**Authors:** Jun Tang, Xiang Wang, Desheng Xiao, Shuang Liu, Yongguang Tao

**Affiliations:** 1 Hunan Key Laboratory of Cancer Metabolism, Hunan Cancer Hospital and The Affiliated Cancer Hospital of Xiangya School of Medicine, Central South University, Changsha, 410078 Hunan China; 2 Key Laboratory of Carcinogenesis and Cancer Invasion (Ministry of Education), NHC Key Laboratory of Carcinogenesis (Central South University), Cancer Research Institute and School of Basic Medicine, Central South University, Changsha, 410078 Hunan China; 3 Department of Thoracic Surgery, Hunan Key Laboratory of Early Diagnosis and Precision Therapy in Lung Cancer, Second Xiangya Hospital, Central South University, Changsha, 410011 China; 4 Department of Pathology, Xiangya Hospital, Central South University, Changsha, 410008 Hunan China; 5 Department of Oncology, Institute of Medical Sciences, National Clinical Research Center for Geriatric Disorders, Xiangya Hospital, Central South University, Changsha, 410008 Hunan China

**Keywords:** CaRNAs, Gene regulation, R-loops, RNA modification, ncRNAs, Cancer

## Abstract

Eukaryotic genomes are prevalently transcribed into many types of RNAs that translate into proteins or execute gene regulatory functions. Many RNAs associate with chromatin directly or indirectly and are called chromatin-associated RNAs (caRNAs). To date, caRNAs have been found to be involved in gene and transcriptional regulation through multiple mechanisms and have important roles in different types of cancers. In this review, we first present different categories of caRNAs and the modes of interaction between caRNAs and chromatin. We then detail the mechanisms of chromatin-associated nascent RNAs, chromatin-associated noncoding RNAs and emerging m^6^A on caRNAs in transcription and gene regulation. Finally, we discuss the roles of caRNAs in cancer as well as epigenetic and epitranscriptomic mechanisms contributing to cancer, which could provide insights into the relationship between different caRNAs and cancer, as well as tumor treatment and intervention.

## Background

In human genomes, approximately 74.7% of genomes can be transcribed to form primary RNAs while 62.1% of genomes generating RNAs will be processed [1]. In those RNAs, some have the ability to interact with chromatin directly or indirectly, called chromatin-associated RNAs (caRNAs). Many noncoding RNAs (ncRNAs) classes can interact with chromatin or bind proteins to form complexes that play key roles in gene regulation. Recently developed technologies such as Mapping RNA-Genome Interactions (MARGI), Chromatin-associated RNA Sequencing (ChAR-seq) and RNA and DNA Interacting Complexes Ligated and sequenced (RADICL-seq) have the ability to detect genome-wide RNA-chromatin interactions, which revealed many chromatin-associated ncRNAs including previously known *Xist* and other novel ncRNAs containing repeat elements and associated transcription start sites [2–5]. These ncRNAs can widely regulate various eukaryotic cellular processes and pathological contexts many diseases, such as cancer [6]. On the other hand, the global RNA interactions with DNA by deep sequencing (GRID-seq) technology demonstrated that a large number of chromatin-enriched RNAs are pre-mRNAs [7]. Recent evidence suggests that some nascent RNAs can act as caRNAs tethering to chromatin accompanied by cotranscriptional processing and RNA modification, which have critical roles in regulatory feedback of transcription and chromatin regulation [8].

In eukaryotic cells, naturally occurring RNAs can basically be divided into protein-coding RNAs and ncRNAs, and ncRNAs can be further divided into several types, including housekeeping ncRNAs and regulatory ncRNAs [9, 10]. However, RNAs with the capability to interact with chromatin are mainly classified into nascent RNAs, long noncoding RNAs (lncRNAs), circular RNAs (circRNAs), small nuclear RNAs (snRNAs), small nucleolar RNAs (snoRNAs), enhancer RNAs (eRNAs), promoter-associated RNAs (paRNAs), antisense RNAs (asRNAs) and repeat RNAs. At first, nascent RNAs either nascent pre-mRNAs or nascent ncRNAs are important caRNAs that can directly bind DNA to form R-loops at transcription sites or act as scaffolds to indirectly interact with chromatin [11, 12]. Next, both lncRNAs and circRNAs are the most common ncRNAs larger than 200 nucleotides in length, and most of them can play transcriptional regulatory roles by interacting with various types of proteins or directly with chromatin and DNA, and they more often regulate chromatin structure and chromatin remodeling by interacting with epigenetic regulators [9]. Then, snoRNAs and snRNAs are housekeeping ncRNAs that only exist in the nucleus and participate in RNA modification and alternative splicing, respectively, moreover, they can form ribonucleoproteins (RNPs) by binding with RNA-binding proteins (RBPs) and then interacting with chromatin [13]. Finally, eRNAs, asRNAs, paRNAs and repeat RNAs are transcribed from genomic-specific sequences, and caRNAs can regulate transcription by binding to DNA sequences or cooperating with RNA modification [14, 15]. Here we mainly discuss the mechanism of these different kinds of caRNAs involved in gene regulation and their roles in tumorigenesis and development. In addition, we also introduce the modification of caRNAs, especially m6A, contributing to epigenetic and epitranscriptomic mechanisms and cancer.

### Mechanisms of caRNAs in gene regulation

#### Types of caRNA-chromatin interactions

In terms of the different locations of caRNAs interacting with chromatin on the genome loci, there are three modes of caRNA-chromatin interactions: *cis*, *trans* and *cis*-*trans* cooperation [16]. Nascent RNAs retained at transcription sites form RNA:DNA heteroduplex (R-loops) that function in transcriptional regulation, which is the most representative interaction mode in *cis*, although there are a few nascent RNAs that can interact with chromatin in *trans* [15–17]. Then, lncRNAs, circRNAs and other chromatin-associated ncRNAs without R-loop formation mainly modulate gene expression in *trans* or *cis*-*trans* cooperation mode, those RNA-chromatin interactions usually occur in noncoding regions such as promoter and enhancer regions of the genome and most of them involve binding to chromatin together with other proteins, even though some lncRNAs can regulate transcription in *cis* by binding DNA to form R-loops [18].

According to the types of combinations between caRNAs and chromatin, the types of caRNAs-chromatin interactions can be basically classified as direct and indirect binding modes. CaRNAs within specific sequences are able to bind both DNA and some proteins such as transcription factors (TFs), RBPs, histone modifiers and chromatin remodelers. Apart from nascent RNAs that directly bind DNA through base complementary pairing, other caRNAs such as paRNAs and repeat RNAs can also directly interact with chromatin [11, 12]. On the other hand, chromatin-associated ncRNAs can indirectly interact with chromatin by acting as a scaffold or chaperone for chromatin remodeling factors and other proteins to form caRNA-protein complexes or RNPs and regulate gene expression [16].

#### The role of chromatin-associated nascent RNAs in transcriptional regulation

**Fig. 1:**
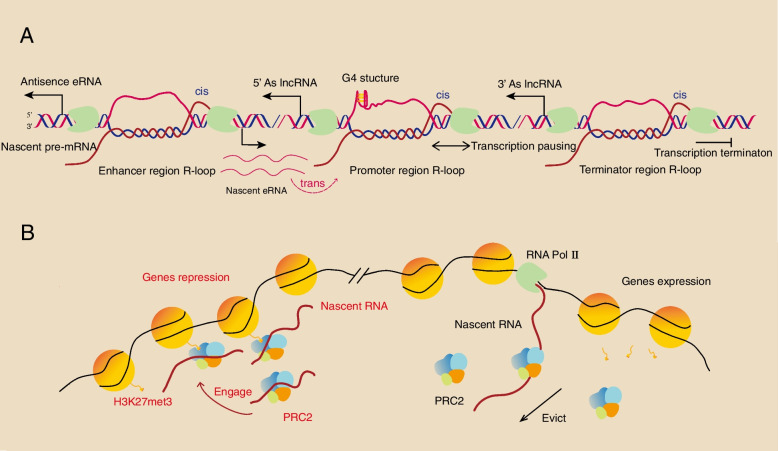
Chromatin-associated nascent RNAs in transcriptional regulation. **A** Nascent caRNAs can form R-loops at promoter, enhancer and termination regions, especially with G4 structures. The promoter region R-loops and terminator region R-loops can contribute to transcription pausing and termination in *cis*, respectively. R-loop formation facilitates antisense lncRNA transcription. Nascent eRNAs can act in *trans* to participate in promoter R-loop formation and gene regulation. **B** Nascent caRNAs can cooperate with epigenetic factors to regulate gene expression. Nascent caRNAs recruit PCR2 to inactive gene regions and promote H3K27me3 spreading, while in active gene regions, nascent caRNAs can continuously evict PRC2 from chromatin. CaRNAs, Chromatin-associated RNAs; G4, G-quadruplex; PRC2, Polycomb repressive complex 2

Nascent RNA including nascent precursor mRNA(pre-mRNA) and nascent ncRNA can both act as caRNAs through *cis* and *trans* mechanisms. The R-loop is formed by nascent RNA: DNA hybrids and a displaced single-stranded DNA during the transcription process especially in highly transcribed regions and repeats such as telomeres [19], which not only affect genome stability and double-strand break (DSB) repair but also regulate transcriptional pausing, termination and antisense transcript synthesis [17, 20–23]. Nascent mRNAs at the site of transcription may form R-loops and act in *cis* to regulate transcription [19]. In eukaryotes especially mammals, nascent RNAs usually form R-loops more easily in GC-skew sequences and repetitive regions [24–26]. A quantitative model of R-loop-forming sequences (QmRLFS) revealed that most R-loops located in G-rich regions and genomic loci in humans are associated with many diseases [15]. Additionally, the presence of G-quadruplex (G4) secondary structure on the displaced single-stranded DNA promotes R-loop formation, which may support to transcriptional regulation and genome instability [27]. R-loops formed in gene promoters contribute to transcription pausing while the GC-skew sequence is important for forming R-loops [26]. However, recent studies have reported that the 5′ end R-loop of the transcripts are associated with low transcription pausing and that the 3′ end R-loop of the transcripts is associated with the defects in transcription termination. Interestingly, the transcripts containing R-loops are generally expressed at higher levels than those without any R-loops [28]. These results indicate that R-loops formed by nascent transcripts act as a kind of expression-promoting factor. In addition, R-loops formation can promote de novo antisense lncRNA synthesis from the genome enhancer region, promoter region and termination region [20].Chromatin-associated nascent RNAs also engage in transcriptional regulation and chromatin accessibility at specially distinct sites in *trans* [29]. One example is that nascent eRNAs form an R-loop that acts in trans and is involved in enhancer-promoter interactions. Such mechanisms mainly include three models: enhancer-associated R-loops (eR-loops), DNA: eRNA-DNA triplex and eRNA-protein-DNA complexes [15] (Fig. 1A).

Another model of nascent RNAs interacting with chromatin involves nascent caRNAs and epigenetic factor cooperation mechanisms. Recent studies have shown a strong relationship between histone modification and transcription. Some histone modifications (e.g., the activating histone marks H3K4me3, H3K27ac, and H3K36me3 and the repressive histone mark H3K27me3) as well as histone modification factors such as polycomb repressive complexe 2 (PRC2) have a broad range of dependence on Pol II. In particular, accumulation of H3K27me3 can be observed after blocking transcription [30]. For instance, nascent RNA can associate with PRC2 participating transcriptional and chromatin state regulation in different ways [31–33]. PRC2 is one of the crucial components of polycomb repressive complexes, and is formed through cPRC1 binding to H3K27me3 and compact chromatin to repress transcription. And another important component is PRC1 which often collaborates with PRC2 in Polycomb chromatin domains to repress transcription. Although PRC1 has more effects on gene repression during early development and in embryonic stem cells (ESCs), PRC2 typically regulates transcription by binding nascent RNA that acts as an RNA bridge to alter H3K27me3 levels in the genome [34]. Recent literature has shown that in human induced pluripotent stem cells (iPSCs) nascent RNA is important for the proper localization of PRC2 on chromatin and affects the differentiation process of iPSCs. In inactive gene regions, nascent RNA, as a bridge, recruits PRC2 to bind chromatin according to the detected chromatin context such as preexisting H3K27me3 of nearby chromatin regions, and then identifies the localization of PRC2 on chromatin and H3K27me3 spreading to repress gene expression. Conversely, nascent Pol II transcripts can also continuously eject PRC2 from local chromatin by releasing RNA-PRC2 complexes during gene activation [31] (Fig. 1B). The above research is consistent with G-tracts or G4 within nascent pre-mRNAs specifically binding PRC2 and removing it from activated genes [32]. On the other hand, the aberrant R-loops at *ENPP2* locus can be unwound by the RNA helicase DExD-Box Helicase 21 (DDX21) and eventually increase RNA pol II processing and transcription, which require the H3K27me3 demethylase JMJD3/KDM6B to recruit and interact with DDX21 at the transcription start site (TSS) [33]. Most of those nascent RNAs interacting with PRC2 are implemented through *cis-acting*, and although many chromatin-associated lncRNAs play an essential role in gene regulation as *cis-* or *trans-acting* [35], the *trans* mode and distant action of the nascent RNA-chromatin interaction and its functions should be further discovered. In addition to histone modifications, the chromatin remodelers also associate with R-loops in specific ways, one example is that BRG1, a subunit of chromatin remodeler SWI/SNF complexes, contributes to the resolution of R-loops and transcription-replication conflicts [36]. Chromatin-associated nascent RNAs mainly participate in the formation of R-loops and impact RNA Pol II transcription, while some chromatin remodelers can also be recruited by nascent RNAs to alter transcription and chromatin state. More novel nascent RNAs and chromatin interaction mechanisms should be further investigated.

#### Chromatin-associated noncoding RNAs have significant functions in gene regulation

The classification of noncoding RNAs is constantly updating and changing as an increasing number of functions of ncRNAs are discovered. Currently, the most classic category and most studied of ncRNAs are mainly s3mall ncRNAs including microRNAs (miRNAs) and ncRNAs over 200 nt in length including circRNAs and lncRNAs [37, 38]. NcRNAs can also be classified into housekeeping ncRNAs and regulatory ncRNAs [10, 39]. In the latter classified ncRNAs, housekeeping snRNA, snoRNA and regulatory ncRNAs including lncRNA, circRNA, paRNA and eRNA can directly or indirectly interact with chromatin, and together make up the chromatin-associated noncoding RNAs regulating gene expression.

**Fig. 2:**
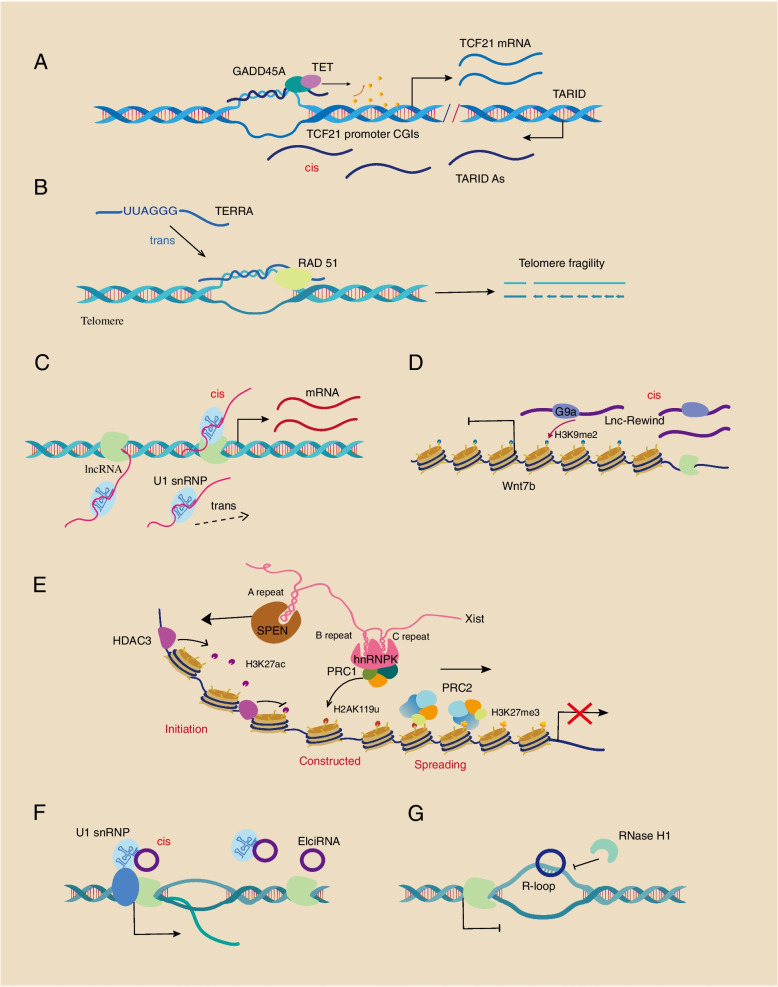
The mechanisms of chromatin-associated noncoding RNAs in gene regulation. **A** and **B** Chromatin-associated lncRNAs directly interact with DNA. **A** Antisense lncRNA TARID directly binds to the TCF21 promoter and is recognized by GADD45A and subsequent TET1, which results in DNA demethylation and TCF21 expression. **B** LncRNA TERRA with a specific sequence forms R-loops in telomeres and is recognized by RAD51, leading to telomere fragility. **C**-**E** Chromatin-associated lncRNAs indirectly interact with DNA or chromatin. **C** LncRNAs bind U1 snRNP to form a complex, which regulates transcription through RNA Pol II both in *cis* and *trans*. **D** LncRNA Rewind can recruit G9a to Wnt7b chromatin, leading to H3K9me2 deposition and gene repression. **E** Xist can bind many proteins, such as SPEN, hnRNP K, PRC1 and PRC2, to mediate transcriptional silencing, which is involved in the initiation, construction and spread of X-chromosome inactivation. **F** and **G** Chromatin-associated circRNAs directly or indirectly interact with DNA. **F** EIciRNAs within specific sequences interact with U1 snRNP at promoters and increase gene expression in *cis*. **G** CircRNA *ci-ankrd52* forms R-loops by directly inserting DNA double strands, which will inhibit transcription, while RNase H1 can resolve R-loops and disarm the transcriptional inhibition effect. TCF21, transcription Factor 21; GADD45A, growth arrest and DNA damage inducible alpha; TET1, tet methylcytosine dioxygenase 1; TCF21, transcription factor 21; hnRNP K, heterogeneous nuclear ribonucleoprotein K

There are large numbers of lncRNAs that have the ability to interact with chromatin in eukaryotes, which generally contain specific sequences or structures to bind DNA, chromatin-associate proteins and chromatin remodelers [23, 40]. Nascent lncRNAs in eukaryotes typically need to be processed, i.e., capped by 7-methyl guanosine (m7G) at their 5′ ends and polyadenylated at their 3′ ends to form mature lncRNAs [41]. The above process is similar to the transcription and processing of nascent mRNA, but current studies have shown that the splicing process of lncRNA is inefficient compared to that of mRNA [42]. A large portion of mature lncRNAs are exported to the cytoplasm, however, numerous lncRNAs are retained in the nucleus through different mechanisms and can provide feedback on transcription and modulate the chromatin state [43]. The mechanisms of lncRNA association with chromatin basically occur via two models, directly or indirectly. One type of lncRNA that directly interacts with chromatin models is lncRNAs that form R-loops at genome loci. Many nascent noncoding RNAs might participate in gene expression by forming R-loops, similarly, some mature lncRNAs can also form R-loops contributing to gene splicing and activation. Chromatin-associated lncRNAs that form R-loops might do function with epigenetic mechanisms in *cis*. In mouse ESCs, the TARID antisense transcript acts as a chromatin-associated lncRNA to form an R-loop at the Transcription Factor 21 (TCF21) promoter, and then epigenetic R-loop reader growth arrest and DNA damage inducible alpha (GADD45A) binds the R-loop and recruits DNA demethylase Tet methylcytosine dioxygenase 1 (TET1) to TCF21 promoter CGIs, which leads local DNA demethylation and TCF21 expression [18] (Fig. 2A). The R-loops formed by lncRNAs also act in *trans*. Telomeric-repeat-containing RNA (TERRA) is a chromatin-associated lncRNA that is usually recruited to chromosome ends in *trans*. Recently, the mechanism of the recruitment of TERRA to chromatin has been revealed, and specific UUAGGG repeats of TERRA and recombinase RAD51 can promote the formation of R-loops by lncRNA TERRA invading into telomere DNA, which contributes to telomere fragility [44] (Fig. 2B). In another instance, the lncRNA APOLO (AUXIN-REGULATED PROMOTER LOOP) in *Arabidopsis* is able to target distant genes and form R-loops, which can decoy the histone modification factor PRC1 component LHP1 to regulate local chromatin 3D conformation and subsequent gene expression [45–47].

The second model of lncRNA-chromatin interaction is indirect, requiring cooperation with RBPs, RNPs and epigenetic factors through specific binding sites in lncRNAs. First, RBPs are extensively present in the transcriptionally active chromatin regions and interact with chromatin, correspondingly, gene promoter regions are RBP-bound hotspots at eukaryotic genomic loci [48, 49]. RBM25 serves as a chromatin-associated RBP contributing to the transcription factor Yin Yang 1 (YY1) mediated enhancer-promoter interaction in HepG2 and K562 cells, which is directly involved in transcriptional activation [48]. Some DNA-binding proteins can also bind directly to lncRNAs through distinct sequences including many TFs such as YY1 [50–52], TEAD [53], Sox2 and p63 [54]. For example, the lncRNA *Linc-YY1* transcribed from the YY1 gene promoter is able to bind YY1 and subsequently evict the YY1-PRC2 complexes at target gene promoters which eventually causing the transcriptional activation [50]. In cerebral ischemia/reperfusion (I/R) injury neurons, lncRNA-GAS can interact with YY1 at the PFKFB3 promoter leading to elevated glycolysis and neuronal apoptosis [51]. A recent study showed that isocitrate dehydrogenase 1 (IDH1) is a novel RBP with the capacity to bind many GA- or AU-rich chromatin-associated lncRNAs [55]. The above cases indicate that some unconventional RBPs can also act as bridges between lncRNAs and chromatin. Additionally, lncRNAs with high U1 snRNP binding sites and poor or no splicing can recruit U1 snRNP, which can then be combined into various genomic regulatory loci and associate with Pol II and chromatin through a *cis-* or *tans-*model [56] (Fig. 2C). Hence, such chromatin-associated lncRNAs act as RNA glue to hold U1 snRNP and RNA Pol II, which can eventually regulate transcription and chromatin state. In adult muscle stem cells (MuSCs), the conserved chromatin-associated lncRNA Lnc-Rewind is able to interact with the chromatin modifier G9a histone lysine methyltransferase in *cis*, which catalyzes the gene repressive histone mark H3K9me2 deposition near Wnt7b loci. Altogether, Lnc-Rewind-mediated repression of the skeletal muscle gene Wnt7b regulates MuSC proliferation and expansion in an epigenetic and chromatin associated manner [57] (Fig. 2D).

Another example of chromatin-associated lncRNAs indirectly binding chromatin to implement gene splicing functions is the well-known dosage compensation mechanism. Dosage compensation is executed by X chromosome inactivation (XCI), which refers to the phenomenon in which female embryonic cells randomly inactivate one of the X chromosomes in order to balance gene dosage in males and females during early mammalian embryonic development [58]. The X-inactivation center (XIC) that can be transcribed to lncRNA Xist is essential and necessary during inactivation [58]. Previous studies have shown that many Xist RNAs might coat the X-chromosome in *cis*, consequently interacting with approximately 1000 genes directly and leading to transcriptional repression [59]. However, the latest research suggests that interaction with only approximately 50 locally confined foci on the inactive X-chromosome by approximately 100 Xist RNAs which trigger more than 1000 genes to become inactive [60]. This process is connected with the recruitment of thousands of proteins mainly including epigenetic factors and associated proteins [61]. Although the exact molecular mechanisms of XCI are still unclear, many studies have shown that the lncRNA Xist might act through epigenetic mechanisms. First of all, lncRNA Xist recruits and binds the transcriptional repressor SPEN through its A-repeat sequence, which associates with many proteins such as histone deacetylase 3 (HDAC3) [62]. Then, activated HDAC3 initiates the gene silencing and XCI by inhibiting the transcriptional activation histone mark acetylated histone H3 Lys27 (H3K27ac). Subsequently, heterogeneous nuclear ribonucleoprotein K (hnRNP K) directly interacts with Xist by its B and C-repeat sequence, which can further recruit the noncanonical PRC1 constructing monoubiquitylates histone H2A Lys119 (H2AK119ub). Additionally, the PRC2 recognizes H2AK119ub and catalyzes the deposition of the transcriptional repressive histone mark H3K27me3 which reinforces gene silencing [5] (Fig. 2E). Therefore, X-chromosome inactivation is a classic model for understanding RNA-chromatin interactions in mammals.

Apart from the lncRNAs that were already introduced above, other chromatin-associated noncoding RNAs such as circRNAs, eRNAs, snRNAs and snoRNAs also have crucial functions in gene regulation. CircRNAs are a class of closed RNA single strands unlike mRNAs and lncRNAs need 5′-capping, 3′-polyadenylation and splicing. Nevertheless, circRNAs also have an essential role in interacting with chromatin and modulating gene transcription [63]. Prior studies reported that circRNAs can interact with chromatin through associating TFs or RNA polymerase II-mediated transcription. Exon-intron circRNAs (EIciRNAs) present in the nucleus interact with U1 snRNP by binding snRNA specific sequences and further associate with RNA Pol II complexes at the promoters to increase gene expression in *cis* [64] (Fig. 2F). Moreover, *ci-ankrd52* can directly insert into DNA double strands forming an R-loop and decelerating RNA Pol II transcription, In addition, RNase H1 binds to the circRNA-DNA R-loop, causing its resolution and promoting transcriptional elongation [65] (Fig. 2G). Furthermore, circRNAs have the ability to interact with TFs in chromatin, similar to chromatin-associated lncRNAs. For example, circRNA circIKBK competitively binds to the NF-κB N-terminus in chromatin, which is the IκBα-binding site. This circRNA mediated mechanism blocks the NF-κB/IκBα negative feedback and promotes BC bone metastasis [66]. The noncoding RNAs transcribed from the gene enhancer region also play a pivotal role in chromatin and gene regulation. TNF-α induced eRNA can interact with bromodomain containing 4 (BRD4) through tandem bromodomains and promote BRD4 binding to H3K27ac- and H4K16ac-modified histone proteins, which further increases the synthesis of eRNAs and the expression of nearby protumor genes [67]. Overall, chromatin associated noncoding RNAs including lncRNAs, circRNAs, and eRNAs, can directly interact with chromatin and DNA to form R-loops or indirectly modulate the chromatin state by binding RBPs, RNPs and epigenetic factors in transcription and gene regulation.

#### The role of m^6^A on caRNAs in chromatin modification and transcriptional regulation

To date, N6-methyladenosine (m^6^A) has been the most abundant dynamic modification that has been found in eukaryotes [68]. The vast majority deposition of m^6^A modification on RNAs depends on METTL3/METTL14/WTAP complexes, which consist of the core protein METTL3 with methyltransferase catalytic activity, adaptor protein METTL14 acting as an allosteric activator and guide protein WTAP recruiting METTL3/METTL14 [69, 70]. Recently, METTL16, METTL5, and ZCCHC4 (zinc finger CCHC-type-containing 4) were discovered as novel discovered m^6^A methyltransferases existing in higher eukaryotes [71–73]. Those RNA methyltransferases are typically called ‘writers’, moreover, there are a few ‘readers’ to recognize m^6^A including YTH domain-containing proteins, hnRNPs, IGF2BPs (insulin-like growth factor 2 mRNA-binding proteins) and ELAV Like RNA Binding Protein 1 (ELAVL1/HuR) as well as some ‘erasers’ such as AlkB Homolog 5 (ALKBH5) and Fat mass and obesity-associated protein (FTO) [70, 74, 75]. The m^6^A modification on different RNAs cooperates with its readers, erasers and writers and has extensive functions in cotranscriptional nascent RNA splicing, mRNA translation, RNA translocation, RNA stability, chromatin state and transcriptional regulation and is related to many diseases especially cancer [74, 76–79]. For instance, the m^6^A modification is associated with the translation of mRNAs in acute myeloid leukemia (AML) cells, and the m^6^A ‘writer’ METTL3 can be recruited by the TF CEBPZ to specific genomic loci promoters, which leads to m^6^A on a few oncogene mRNAs especially SP1 and promotes translation as well as regulates c-MYC expression and AML cell growth [80].

**Fig. 3:**
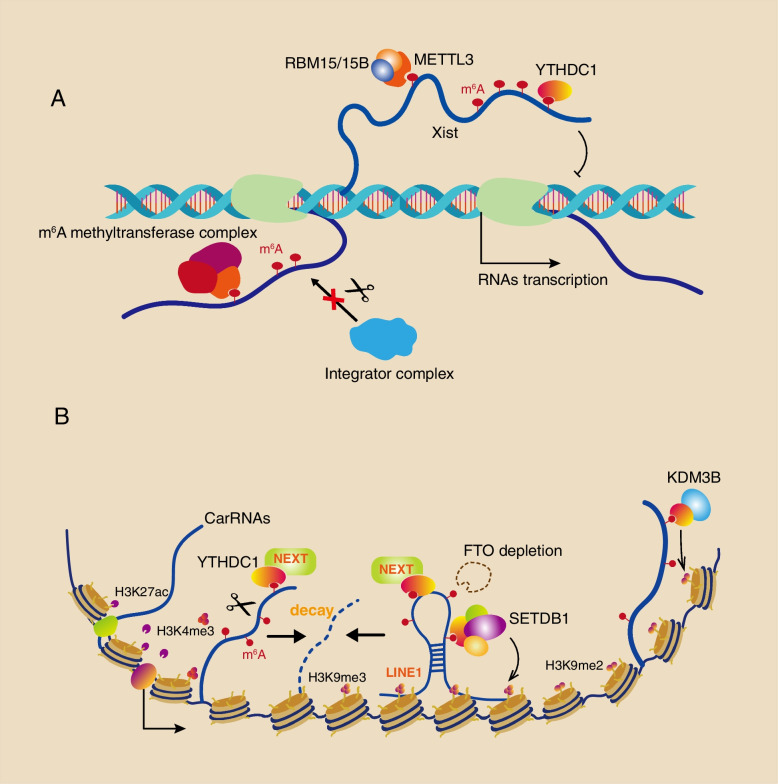
m^6^A on caRNAs regulates transcription and gene expression. **A** The m^6^A methyltransferase complex can recognize m^6^A on nascent RNAs (nascent eRNAs, upstream promoter RNAs, etc.) to ensure mRNA transcription while inhibiting premature termination by the integrator complex. Moreover, Xist-mediated X chromosome gene silencing requires m^6^A modification and YTHDC1 reorganization. **B** m^6^A on caRNAs crosstalk with epigenetics in gene regulation. m6A on carRNAs such as eRNAs and repeat RNAs can be recognized by YTHDC1 and NEXT, resulting in carRNA degradation, while demethylated carRNAs recruit histone modifiers and the TF YY1 to activate transcription. The m^6^A eraser FTO and reader YTHDC1 both play important roles in repeat RNA LINE1-mediated gene repression, and FTO depletion causes higher m^6^A levels of LINE1 and leads to recruitment of YTHDC1, which not only recruits the NEXT complex to accelerate LINE1 decay but also recruits SETDB1 and cooperating proteins to deposit the repressive histone mark H3K9me3. However, YTHDC1-recruited KDM3B can decrease the H3K9me2 level to promote gene expression. m6A, N6-methyladenosine; YTHDC1, YTH domain containing 1; CarRNAs, Chromatin-associated regulatory RNAs; NEXT, Nuclear exosome targeting; LINE1, Long interspersed nuclear elements 1; ERNAs Enhancer RNAs; YY1, Yin Yang 1; SETDB1, SET domain bifurcated histone lysine methyltransferase 1

Many studies have demonstrated that the m^6^A on chromatin-associated nascent RNAs, chromatin-associated regulatory RNAs (carRNAs) and chromatin-associated lncRNAs all have important roles in transcriptional regulation by interacting with m^6^A ‘readers’, histone modifiers and transcription factors [81–84]. First, m^6^A on nascent mRNAs can impact nascent RNA synthesis. The present study reveals that METTL3/METTL14/WTAP complex-mediated m^6^A modification of nascent mRNAs, eRNAs and upstream promoter RNAs can protect premature termination by integrator complexes while increasing nascent RNA transcription [83, 85] (Fig. 3A). On the other hand, m^6^A modification of nascent RNAs and repeat RNAs is able to impact the formation of R-loops thereby influencing transcription termination and heterochromatic RNA synthesis. One example is that the depletion of the METTL3 will dramatically decreases R-loop formation and perturbs transcription termination, and METTL3-deposited m^6^A on nascent RNAs can increase R-loop formation in gene terminator regions and promote transcription termination [86]. A recent study demonstrated that the m^6^A on major satellite repeats (MSRs) can also facilitate the R-loop formation, which promotes the MSR transcription and stabilizes the heterochromatin [14].

More importantly, m^6^A on chromatin-associated RNAs can crosstalk with epigenetics to regulate transcription and gene expression [87, 88]. In mESCs, carRNAs including paRNAs, eRNAs and repeat RNAs are modified by m^6^A through METTL3, and then the m^6^A ‘reader’ YTHDC1 identifies nuclear exosome targeting (NEXT) complexes, resulting in carRNA degradation. Conversely, when demethylated carRNAs bind to chromatin, they can recruit histone modifiers to add transcriptional activators of H3K27ac and H3K4me3, then recruit TF YY1 and repel PRC2, ultimately promoting chromatin opening and gene transcription [82] (Fig. 3B left). Correspondingly in the same cell lines, the depletion of the m^6^A ‘eraser’ FTO can increase m^6^A abundance in long interspersed nuclear element 1 (LINE1) RNA, and then m^6^A recruits YTHDC1 to chromatin, further leading the local chromatin closure through the following two mechanisms: (i) YTHDC1 recruits NEXT complexes and causes LINE1 RNA decay inhibiting the deposition of active histone marks H3K4me3/H3K27ac; (ii) YTHDC1 recruits histone modifier SET domain bifurcated histone lysine methyltransferase 1 (SETDB1) and cofactor tripartite motif containing 28/KRAB-associated protein (TRIM28/KAP1), which adds the repressive histone marks H3K9me3 [75]. Another study showed that YTHDC1 depletion causes the reactivation of silenced retrotransposons including LINE1, which also requires SETDB1-mediated H3K9me3 [89] (Fig. 3B middle). SETDB1-YTHDC1 cooperative model likewise takes place in the intracisternal A particle (IAP)-type family of endogenous retroviruses retrovirus transcription [90]. Similarly, in mouse ESCs 2-cell (2C), m^6^A on LINE1 is recognized by YTHDC1, then LINE1 acting as a scaffold recruits TRIM28/KAP1 and NCL to chromatin and deposits the repressive histone mark H3K9me3 on 2C-related retrotransposons, which eventually inhibits retrotransposons transcription [91] (Fig. 3B middle). Another histone modifier Histone lysine demethylase 3B (KDM3B) can also be recruited to m^6^A -associated chromatin regions by YTHDC1, however, this decreases the H3K9me2 level and promotes gene expression [92] (Fig. 3B right). Therefore, the METTL3, FTO and YTHDC1-mediated carRNA m^6^A modification in cooperation with histone modifier mechanisms ultimately regulate the repeat RNAs and heterochromatin formation during embryonic development.

Finally, m^6^A on chromatin-associated lncRNAs such as Xist is critical for transcriptional regulation. One study showed that RNA-binding motif protein 15 (RBM15) and its paralog RBM15B recruit WTAP/METTL3 complexes to specific sites in Xsit which deposits m^6^A modifications on Xist. This leads YTHDC1 to preferentially recognize m^6^A modification and is required for Xist-mediated gene silencing [84] (Fig. 3A). The latest study revealed the mechanism of the YTHDC1-Xist interaction, m^6^A can be easily deposited in conserved AUCG tetraloop hairpin regions of the Xsit A-repeats and YTHDC1 recognizes it in a single-stranded conformation [93].

In another study, Xist was identified as the direct downstream target of METTL14 resulting in the m^6^A modification, and then the ‘reader’ YTHDF2 can recognize m^6^A-methylated Xist, which contributes to Xist degradation [94]. In addition, circRNAs retained in the nucleus may be involved in different nuclear processes, such as transcriptional and chromatin regulation, by interacting with TFs or RNPs, as illustrated in the last chapter, however, whether m^6^A modification of nuclear circRNAs can also regulate transcription by interacting with chromatin deserves further study. From another perspective, circRNAs may interact with m^6^A ‘writers’, ‘readers’ or ‘erasers’ that indirectly influence the m^6^A level of RNAs and gene expression [95].

In summary, chromatin-associated RNAs can act as targets of m^6^A regulation to modulate chromatin accessibility or closure, which further regulates transcription, heterochromatin formation and gene expression. Even though an increasing number of m^6^A-mediated caRNA functions have been found, including carRNA decay, caRNA-epigenetic interactions, etc., the m^6^A on caRNAs as well as its roles in gene regulation and the crosstalk between epitranscriptomic and epigenetic mechanisms under different contexts should be deeply researched.

### The roles of caRNAs in cancer

#### Aberrant R-loops formed by chromatin-associated RNAs in cancer

The overabundance of R-loops is associated with many diseases including human neurological disorders, motor neuron disorders and different types of cancers [96–98]. In particular, the aberrant accumulation of DNA: RNA hybrids cause DNA damage and genome instability are related to many oncogene mutations and dysregulation. In this section, we mainly discuss the relationship between R-loops formed by caRNAs and human cancers from the following three perspectives: (i) R-loop-associated genomic instability and DNA damage have dual roles in cancer (ii) R-loop formation can affect transcription to modulate cancer progression and (iii) the R-loop-associated mechanism provides a novel insight into cancer targeted therapies.

**Fig. 4:**
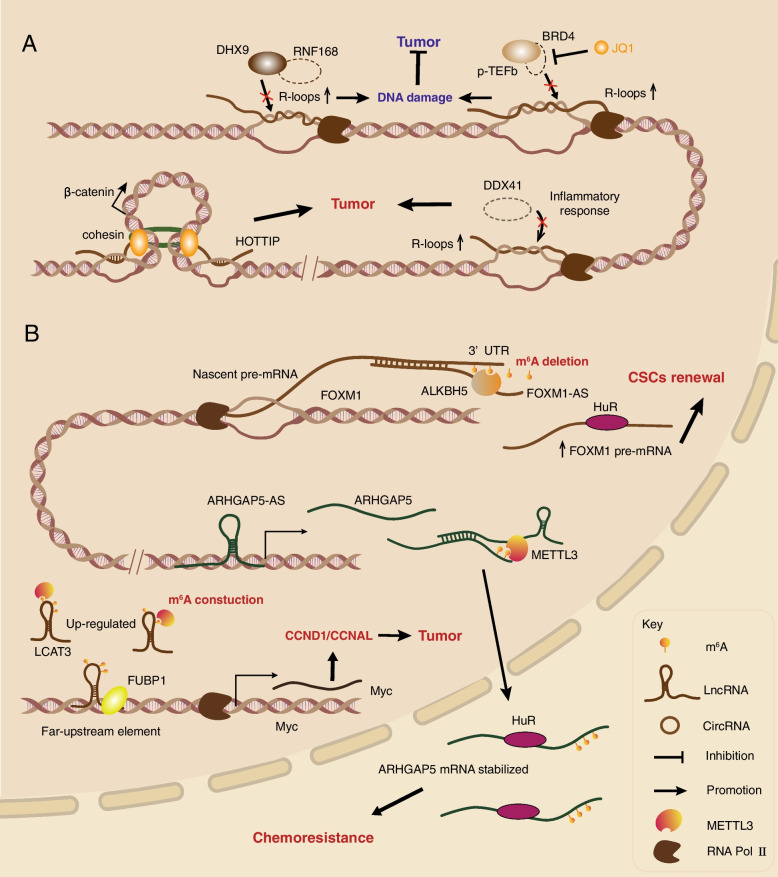
CaRNAs form R-loops and the m^6^A modification on caRNAs in cancer. **A** The accumulation of R-loops resulted in genomic instability, and DNA damage has dual roles in cancer. The deletion of the DNA damage response protein RNF168 or transcription coactivator BRD4 blocks the unwinding of DNA and RNA heterozygous strands on the genome, leading to the accumulation of R-loops on the cancer cell genome and tumor repression. The BRD4 inhibitor JQ1 can also lead to similar accumulation of R-loops. Mutation of the tumor suppressor DDX41 causes aberrant R-loop accumulation, and genomic instability stimulates the inflammatory response and AML progression. Similarly, lncRNA *HOTTIP*-formed R-loops can recruit the CTCF/cohesin complex at TAD boundaries, which promotes Wnt/β-catenin transcription and accelerates leukemogenesis as well as AML development. **B** ALKBH5 can specifically decrease the level of m^6^A in the 3’UTR of FOXM1 pre-mRNA, while As-FOXM1 promotes the interaction between ALKBH5 and FOXM1 pre-mRNA, which increases FOXM1 expression as well as GSC self-renewal and tumorigenesis. Similarly, As-ARHGAP5 can not only promote the transcription of ARHGAP5 but also induce METTL3 to deposit m^6^A modifications on ARHGAP5 mRNA, increasing the stability of ARHGAP5 mRNA in the cytoplasm and promoting the chemotherapy resistance of gastric cancer. Notably, HuR is involved in mRNA regulation in both the nucleus and cytoplasm. Another lncRNA, LCAT3, can be directly recognized by METTL3 and modified by m^6^A to increase stability and be upregulated, then it can bind to the far upstream elements of MYC together with FUBP1, leading to MYC activation and proliferation, invasion and metastasis of lung cancer cells. RNF168, Ring Finger Protein 168; BRD4, Bromodomain Containing 4; ELAVL1/HuR, ELAV Like RNA Binding Protein 1; DDX41, Dead box helicase 41; AML, Acute myeloid leukemia; TAD, Topologically associated domain; ALKBH5, AlkB Homolog 5; METTL3, Methyltransferase like 3; FOXM1, Forkhead Box M1; ARHGAP5, Rho GTPase Activating Protein 5; LCAT3, Lung Cancer Associated Transcript 3; FUBP1, Far Upstream Element Binding Protein 1

First and foremost, the transcription-replication conflict-caused R-loop accumulation can increase DNA damage and genomic instability, which are correlated with oncogenic events in different human cancers. In cancer cells, oncogene mutation and activation can initiate abnormal transcriptional programs, and since same time cancer cells are at a high replication state, this will result in transcription-replication conflicts (TRCs) [96]. Replication stress caused by transcription-replication conflicts typically acts as a primary source of R-loop accumulation during oncogene activation [99]. Notably, TRC and R-loop accumulation can increase DNA damage, which can suppress tumor cells [100]. For example, in cancer cells, the breast cancer type 1/2 susceptibility protein (BRCA1/2) mutation or deficiency can lead to R-loop accumulation and subsequent genomic instability and DNA double-strand breaks (DSBs) [101–103]. A recent study noted that elevated R-loops and subsequent DNA damage, senescence and cell death will suppress tumor cells in BRCA1/2-mutant breast and ovarian cancer, and the detailed mechanism is related to the DNA damage response protein Ring Finger Protein 168 (RNF168) deficiency, which disrupts the recruitment of DExH-Box Helicase 9 (DHX9) at R-loop genome loci and resolution of R-loops [104] (Fig. 4A). Such RNF168 deficiency-induced R-loop accumulation can heighten the sensitivity of cancer cells to G4/R-loop-stabilizing drugs such as PARP inhibitors (PARPi), pyridostatin, and irradiation. A similar example is the loss or inhibition of the transcriptional coactivators BRD4, which can decrease the transcription of the DNA damage response protein DNA Topoisomerase II Binding Protein 1 (TopBP1) and lead to accumulation of R-loops, resulting in DNA damage and oncogenic cell apoptosis [105] (Fig. 4A). In Ewing sarcoma cells, the fusion of EWS RNA binding protein 1 (EWSR1) and Friend leukemia integration 1 transcription factor (FLI1) EWS-FLI1 can increase transcription, replication stress and R-loop formation and lead to blocked BRCA1-mediated repair after DNA damage [106]. This finding is consisting with Ewing sarcoma cells’ high sensitivity to chemotherapy such as PARP1 inhibitors and normal cells containing wild-type EWSR with efficient DNA repair. R-loops can also associate with epigenetic regulators to participate cancer progression in prostate cancer cells, the ATP-dependent chromatin remodeling INO80 complexes have been found to remove the R-loops from chromatin and prevent transcription-replication conflicts, which protects cancer cells from genotoxins and subsequent DNA damage, ultimately promoting unlimited cancer cell proliferation and existence [107].

Similarly, R-loop formation causes genomic instability, which can inhibit tumor growth in human cancers. It is well known that one of the major hallmarks of cancer is genome instability [108]. A few studies have reported that R-loop formation is associated with cancer genome instability. Adenosine deaminase acting on RNA 1(ADAR1) is the enzyme that participates in adenosine-to-inosine RNA editing and consists of two isoforms: ADAR1p150 in the cytoplasm and ADAR1p110 in the nucleus. A recent study found that pro-oncogenic ADAR1p110 can convert A-C mismatches to I-C matched base pairs and increase RNase H2 activity, promoting the resolution of telomeric R-loops, which is required for the continued proliferation of telomerase-positive cancer cells. In turn, the inhibition or depletion of ADAR1p110 suppresses tumor proliferation by R-loop accumulation, genomic instability and apoptosis [109]. This research shows that telomeric R-loop formation plays an essential role in telomeric stability and human cancers. Another appropriate example is the chromatin-associated lncRNA TERRA, which has been introduced in the mechanism of caRNAs in gene regulation section of this paper., lncRNA TERRA-formed R-loops are able to recruit the DNA damage repair-associated protein BRCA1 and subsequent epigenic repressive regulators at CpG-rich TERRA promoters to repress TERRA transcription and maintain telomer integrity, while TERRA upregulation caused by the depletion of BRCA1 leads to R-loop accumulation at the TERRA promoter and telomers, which results in increased replication stress and telomer DNA damage as well as telomer instability [110]. Many studies have indicated that R-loop-induced DNA damage and genomic instability are related to cancer suppression. However, aberrant R-loops accumulation caused by tumor suppressor loss may promote cancer progression. The mutant of tumor suppressor Dead box helicase 41 (DDX41) can block the unwinding of RNA–DNA hybrids, which results in aberrant R-loop accumulation and genomic instability, while those conditions can ultimately increase the inflammatory response and development of familial AML [111] (Fig. 4A).

On the other hand, R-loop-associated transcriptional regulation also plays essential roles in tumorigenesis and cancer progression. Aberrant R-loop accumulation localized at the TSS can promote tumorigenesis by affecting associated gene transcription. BRCA1 mutation causes nascent RNA-formed R-loop accumulation at the luminal gene and its enhancer region in luminal progenitor cells, which inhibits the enhancer-promoter interactions and transcriptional activation and subsequent primary luminal progenitor cell differentiation into mature luminal cells, resulting in oncogenic transformation and basal-like tumor formation [112]. Chromatin-associated lncRNAs can also participate in R-loop formation and regulate transcription in AML. The lncRNA *HOTTIP* directly interacts with CTCF-binding sites (CBSs) to form R-loops and then recruits CTCF/cohesin complexes and R-loop-associated factors, which reinforces the CTCF boundary and promotes the formation of the β-catenin topologically associated domain (TAD). The above *HOTTIP* and CTCF/cohesin mediated R-loop formation at TAD boundaries drives canonical Wnt/β-catenin transcription and ultimately facilitates leukemogenesis and AML development [113] (Fig. 4A).

Finally, R-loops may contribute to targeted cancer therapies. Previous examples of R-loops being involved in the cancer proliferation and progression suggest that targeting transcription-replication conflicts and R-loop formation-associated oncoproteins, such as RNF168, INO80, BRD4, DDX41 and EWS-FLI1, and promoting or inhibiting them may heighten the sensitivity of cancer cells to DNA damage, genomic instability and the immune response, which could ultimately inhibit tumor proliferation to achieve well targeted therapies. It has been demonstrated that cancer-targeted therapeutic drugs including BET inhibitors (BETis) and histone deacetylase inhibitors (HDACis) are associated with replication stress and R-loops accumulation [114–116]. Hence, R-loop formation related targeted therapy mechanisms and novel target molecules should be further discovered, and such insight can provide more effective targeted treatment for cancer. Generally, caRNA-formed R-loops can function through DNA damage, genomic instability and transcriptional regulation in cancer. Apart from the above models, there might be other aspects of R-loops associated with cancers such as epigenetic and epitranscriptomic regulation [117, 118].

#### Emerging roles of caRNAs related with m^6^A modification in cancer

Numerous previous studies have shown that cytoplasmic mRNAs and noncoding RNAs with m^6^A modification can be associated with many disease occurrence mechanisms including cancer by m^6^A writers-, erasers- and readers-mediated RNA fate decisions and translation regulation [77, 119–122]. For instance, in ovarian cancer cells, the upregulation of m^6^A reader YTHDF1 specifically binds m^6^A-modified Eukaryotic Translation Initiation Factor 3 Subunit C (EIF3C) mRNA and facilitates its translation. Then the increased EIF3C, a subunit of the protein translation initiation factor EIF3, can promote ovarian cancer tumorigenesis and metastasis [123]. Correspondingly, the depletion of the m^6^A methyltransferase METTL3 in macrophages can decrease the YTHDF1-mediated translation of Sprouty Related EVH1 Domain Containing 2 (SPRED2), impairing the inhibition of ERK by SPRED2, which eventually leads the activation of NF-κB and Signal Transducer and Activator of Transcription 3 (STAT3) as well as facilitates tumor growth and metastasis [124]. On the other hand, m^6^A readers mediate RNA decay, which is associated with tumorigenesis, YTHDF2 is observed to be overexpression in glioblastoma cells and plays an important role in cholesterol metabolism-associated liver X receptor a (LXRa) and HIVEP zinc finger 2 (HIVEP2) mRNA decay in a m^6^A-dependent manner, which finally causes cholesterol dysregulation and promotes the glioblastoma cell proliferation, invasion and tumorigenesis [125].

More importantly, with an increasing number of m^6^A readers and associated proteins being discovered, especially m^6^A -associated factors located in the nucleus and chromatin, the functions of caRNAs associated with m^6^A modification are gradually expanding. Among them, there are a few caRNAs related to m^6^A modification involved in cancer progression and tumorigenesis (Fig. 4B). A significant body of work has provided a novel mechanism of m^6^A on chromatin-associated nascent RNAs in glioblastoma stem-like cells (GSCs). Researchers have found that the m^6^A demethylase ALKBH5 can specifically recognize the 3′ UTR of nascent Forkhead Box M1(FOXM1) pre-mRNA, resulting in a decrease in m^6^A level on FOXM1 pre-mRNA, and an increase in HuR-mediated FOXM1 expression, which leads to GSC self-renewal and tumorigenesis. The lncRNA FOXM1 antisense transcript is able to facilitate the interaction between nascent FOXM1 pre-mRNA and ALKBH5 [126]. Likewise, Rho GTPase activating protein 5 (ARHGAP5) antisense transcript lncRNA ARHGAP5-AS1 can not only promote ARHGAP5 transcription by interacting with chromatin but also recruit METTL3 to ARHGAP5 mRNA, elevating the m^6^A modification level and stability of ARHGAP5 mRNA in the cytoplasm, which ultimately increases the chemoresistance of gastric cancer (GC) [127]. Another lncRNA GATA3-AS, antisense transcript of tumor-suppressing gene GATA Binding Protein 3 (GATA3), can also recruit the m^6^A writer KIAA1429 to the 3′ UTR of GATA3 pre-mRNA and deposit m^6^A methylation, which inhibits the interaction between GATA3 pre-mRNA and HuR, leading to the subsequent pre-mRNA degradation [128]. Interestingly, the cytoplasmic m^6^A-modified transcripts play contrasting roles in mRNA stabilization by interacting with the RBP HuR, hence further mechanisms of m^6^A and HuR-mediated RNA regulation should be investigated. The above examples show that lncRNAs indirectly associate with m^6^A modification by cooperating with mRNA and nascent pre-mRNA. Additionally, chromatin-associated lncRNAs can be directly deposited on m^6^A and subsequently participate in oncogene regulation and cancer progression. For example, the novel lncRNA lung cancer associated transcript 3 (LCAT3), is stabilized and upregulated in lung adenocarcinomas due to METTL3-mediated m^6^A modification, and then LCAT3 recruits Far Upstream Element Binding Protein 1 (FUBP1) to the MYC far-upstream element sequence, which leads to MYC activation and lung cancer cell proliferation, invasion and metastasis [129]. Although previous studies have shown that the communication between m^6^A and epigenetic regulation plays an important role in gene regulation, there are still few studies on its function in tumorigenesis and development. The newest study found that the RNA m^6^A level in esophageal squamous cell carcinoma tissues is significantly higher than that in paracarcinomic tissue. Further studies have shown that RNA m^6^A can negatively regulate the DNA methylation of esophageal cancer through FMR1 Autosomal Homolog 1 (FXR1) and TET1 and play an important role in the development of cancer [130]. However, other types of caRNAs such as some circRNAs, snRNAs, snoRNAs and carRNAs seem to rarely associate with m^6^A modification in cancer. Further studies should provide insight into the elusive and unknown association between m^6^A-modified caRNAs and cancer.

#### Noncoding RNAs interact with chromatin in cancer

Noncoding RNAs, especially lncRNAs, miRNAs and circRNAs, can participate in and modulate many biological processes, including RNA transcription, translation, and alternative splicing, contributing to the regulation of gene expression and cancer [41, 131, 132]. There are many noncoding RNAs, including some lncRNAs, circRNAs, eRNAs and snoRNAs, that can directly or indirectly interact with chromatin, which regulates gene transcription and subsequent physiological processes associated with the progression of various human cancers and tumorigenesis (Table 1).

**Table 1:** Selected caRNAs linked to human cancers

Type	CaRNA	Interaction mode	Function	References
lncRNA	*HOTTIP*	Forms R-loops at CBSs and recruits CTCF/cohesin complex	Facilitates leukemogenesis and AML development	[113]
	MaTAR25	Interacts and recruits transcriptional coactivator PURB to Tensin1 promoter	Increases Tensin1 transcription and BC cells proliferation, invasion and migration	[133]
	RP11-19E11.1	Directly interacts with TF E2F1	Attenuates DNA damage and apoptosis to aid BC cells survival	[134]
	ANRIL	Interacts with ATR and stabilizes it by deubiquitin	Guarantees homologous recombination repair and lung cancer cells survival	[135]
	LINC00839	Recruits transcriptional activator Ruvb1/KAT5 complex and helps to deposit H4K5ac and H4K8ac at NRF1 promoter	Increases NRF1 expression and promotes CRC OXPHOS and procession	[136]
	BlackMamba	Binds and recruits LSH to cytoskeleton formation and inflammation-related gene promoters	Maintains ALCL neoplastic phenotype and cell growth	[137]
	LINC-PINT	Interacts with PRC2 at EGR1 and ITGA3 promoters	Silences EGR1 and ITGA3 gene to repress cancer cells migration	[138]
	LCAT3	LCAT3 Recruits FUBP1 to the MYC far-upstream element sequence	Activates MYC and promotes lung cancer cells proliferation and metastasis	[129]
asRNAs	ARHGAP5-AS1	Recruits METTL3 to ARHGAP5 mRNA to elevate the m^6^A level	Stabilizes ARHGAP5 mRNA and increases chemoresistance of GC	[127]
	GATA3-AS	Recruits m^6^A writer KIAA1429 to the GATA3 3’UTR	Leads to GATA3 pre-mRNA degradation and promotes tumor progression	[128]
	FOXD2-AS1	Recruits EZH2 to the CDKN1B promoter and induces the deposition of H3K27me3	Silences CDKN1B and promotes HCC proliferation	[139]
	Linc-ASEN	Interacts with UPF1-PRC1/PRC2 complex and binds p21 gene 3’UTR	Silences p21 gene and retards CRC and BC cells senescence	[140]
circRNA	circIPO11	Recruits TOP1 to GLI1 promoter	Activates GLI1 transcription and promotes the HCC progression	[141]
	circMRPS35	Binds to FOXO1 and FOXO3a gene promoters and recruits KAT7 depositing H4K5ac	Upregulates p21 and p27 and downregulates Twist1 and E-cadherin and inhibits GC proliferation and invasion	[142]
	circGSK3B	Interacts with EZH2 at RORA promoter and inhibits the deposition of H3K27me3	Upregulates RORA and attenuates GC growth, invasion and metastasis	[143]
	circNCOR1	Recruits hnRNPL to the SMAD7 promoter and meanwhile inserts in DNA to form R-loops	Upregulates SMAD7 and decreases BC cells growth and LN metastasis	[144]
snoRNA	SNORA73	Combines poly PARP1 and DKC1/NHP2 to form a snoRNP at DNA damage genomic loci	Blocks DNA damage repair and promotes AML differentiation	[145]
nascent RNA	FOXM1 pre-mRNA	Recognized by ALKBH5 and with lower m^6^A	Promotes FOXM1 expression and GSCs renewal and tumorigenesis	[126]

**Fig. 5:**
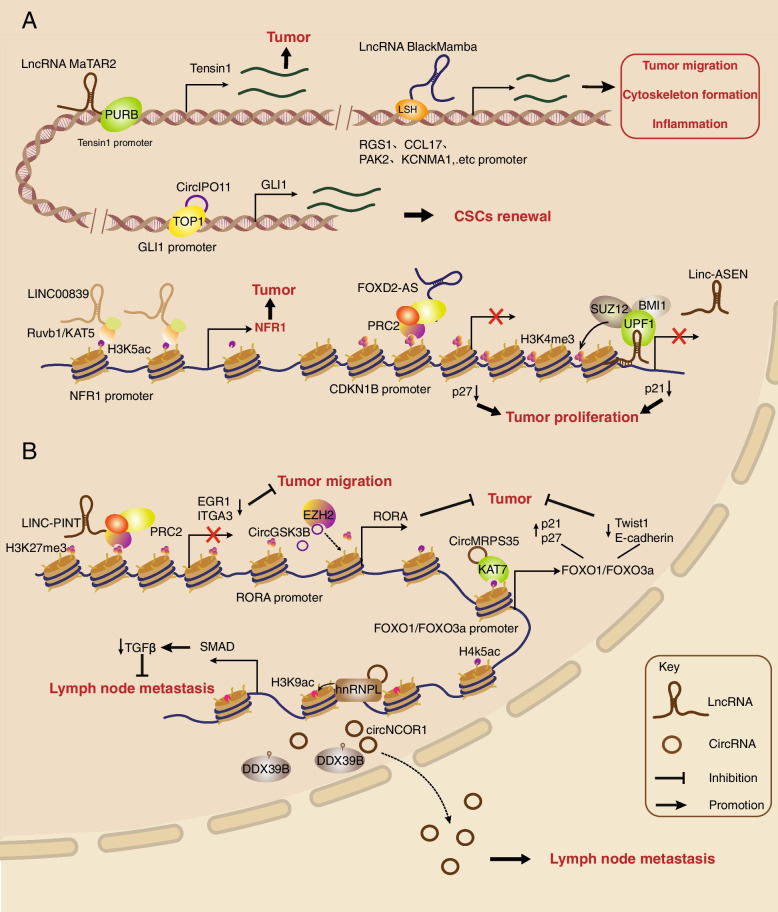
Chromatin-associated noncoding RNAs in cancer. ncRNAs have dual roles in cancer by interacting with intranuclear TFs, epigenetic regulators, transcriptional coactivators and DNA/RNA helicases on chromatin. **A** Chromatin-associated ncRNAs contribute to tumor progression. LncRNA MaTAR2 recruits the transcriptional coactivator PURB at the Tensin1 promoter to promote transcription and BC proliferation, invasion and migration. LncRNA BlackMamba can bind and recruit LSH to the promoter regions of tumor migration-related genes such as Regulator of G Protein Signaling 1 (RGS1), Thymus and activation-regulated chemokine (TARC), P21 (RAC1) Activated Kinase 2 (PAK2) and Potassium Calcium-Activated Channel Subfamily M Alpha 1 (KCNMA1) to promote transcription and ultimately maintain the ALCL phenotype and growth. CircIPO11 recruits TOP1 to the GLI1 promoter to activate transcription and promotes the self-renewal of liver CSCs. At the chromatin level, lncRNA LINC00839 recruits the transcriptional activator Ruvb1/KAT5 complex to the NRF1 promoter to deposit activating histone marks H4K5ac and H4K8ac and promote OXPHOS and CRC processes. Some caRNAs can also recruit repressive histone marks. lncRNA FOXD2-AS1 can induce EZH2 to the CDKN1B promoter and construct H3K27me3 to silence CDKN1B, leading to HCC proliferation. Similarly, lncRNA Linc-ASEN can interact with the UPF1-PRC1/PRC2 complex to silence p21 and delay tumor cell senescence. **B** Chromatin-associated ncRNAs inhibit tumor progression. LINC-PINT recruits PRC2 to the EGR1 and ITGA3 gene loci and induces their silencing, which ultimately inhibits tumor migration. CircMRPS35 recruits KAT7 to the FOXO1 and FOXO3a promoters, leading to upregulation of p21 and p27 and downregulation of Twist1 and E-cadherin, thereby inhibiting GC proliferation and invasion. CircGSK3B can directly bind EZH2 to block its deposition on the RORA promoter, which causes the upregulation of RORA and finally inhibits GC progression. CircNCOR1 recruits hnRNPL to the SMAD7 promoter, promoting SMAD7 transcription and inhibiting the TGFβ/SMAD signaling pathway, which ultimately inhibits BC growth and lymph node metastasis. However, the SUMOylated DDX39B-mediated abnormal export of circNCOR1 from the nucleus to the cytoplasm weakens the tumor-inhibiting effect of circNCOR1. PURB, Purine rich element binding protein B; NRF1, Nuclear Respiratory Factor 1; CRC, Colorectal cancer; OXPHOS, Oxidative phosphorylation; RGS1, Regulator of G Protein Signaling 1; CCL17, Thymus and activation-regulated chemokine, TARC; PAK2, P21 (RAC1) Activated Kinase 2; KCNMA1, Potassium Calcium-Activated Channel Subfamily M Alpha 1; ALCL, Anaplastic large cell lymphoma; LSH, ymphoid-specific helicase; EZH2, Enhancer of zeste homolog 2; CDKN1B, Cyclin Dependent Kinase Inhibitor 1B; EGR1, Early Growth Response 1; ITGA3, Integrin alpha 3; TOP1, Topoisomerase 1; GLI1, GLI Family Zinc Finger 1; CSCs, Cancer stem cells; HCC, Hepatocellular carcinoma; KAT7, Lysine Acetyltransferase 7; Twist1, Twist Family BHLH Transcription Factor 1; GC, Gastric cancer

Many oncogenic chromatin-associated lncRNAs have the capability to interact with many transcriptional regulators, including TFs, DNA/RNA-binding proteins and epigenetic factors (Fig. 5A). At the Tensin1 promoter region, the chromatin-associated lncRNA MaTAR25 can recruit and specifically interact with purine rich element binding protein B (PURB), a transcriptional coactivator and sequence-specific DNA-binding protein that also has RNA-binding ability, which upregulates the focal adhesion complex component Tensin1 linking the intracellular cytoskeleton and extracellular matrix, ultimately resulting in breast cancer cell proliferation, invasion and migration [133]. Likewise, lncRNA RP11-19E11.1 is an upregulated caRNA and is related to poor prognosis in patients with basal breast cancer. Further study showed that RP11-19E11.1 can directly interact with TF E2F Transcription Factor 1 (E2F1), which is essential for inhibiting the DNA damage response and apoptosis and maintaining cancer cell proliferation and survival [134]. Such a DNA damage response linked to caRNA and cancer also occurs with the lncRNA ANRIL. The caRNA ANRIL binds with the DNA repair protein ATR and increases its stability by arresting ubiquitination-mediated degradation, ensuring homologous recombination repair in lung cancer cells under DNA damage and cell survival as well as cancer resistance [135]. Moreover, caRNAs can participate in transcriptional regulation and cancer progression by interacting with epigenetic factors. The latest research found that lncRNA LINC00839 acts as an RNA scaffold to recruit transcriptional activator RuvB-like AAA ATPase 1/Lysine Acetyltransferase 5 (Ruvb1/KAT5) complexes to the Nuclear Respiratory Factor 1 (NRF1) promoter region and increases H4K5ac and H4K8ac levels at the promoter, which increases NRF1 expression and finally promotes colorectal cancer (CRC) cell OXPHOS and CRC progression [136]. In anaplastic large cell lymphoma (ALCL), the STAT3-regulated caRNA BlackMamba has the capability to bind and recruit the chromatin remodeling protein lymphoid-specific helicase (LSH/HELLS) to the promoter region of genes related to tumor migration, cytoskeleton formation and inflammation, which increases associated protein expression and maintains the lymphoma kinase-negative (ALK-) ALCL neoplastic phenotype and cell growth [137]. The above lncRNAs interacting with chromatin are all related to associated gene upregulation of transcription, and some lncRNAs contribute to transcription silencing in cancer development. For instance, lncRNA FOXD2-AS1 can recruit PRC2 submit Enhancer of zeste homolog 2 (EZH2) to the Cyclin Dependent Kinase Inhibitor 1B (CDKN1B) promoter region and deposit H3K27me3, which silences CDKN1B gene transcription, leading to the downregulation of CDKN1B-coded cyclin-dependent kinase inhibitor p27 and proliferation of hepatocellular carcinoma [138]. In particular, lncRNA is able to directly bind to the DNA double strand through its specific sequence and connect to other regulators, accelerating cancer progression. LncRNA Linc-ASEN can bind to the p21 gene 3’UTR within a specific sequence while interacting with UPF1-PRC1/PRC2 complexes that silence p21 gene transcription and subsequently retard tumor cell senescence [139].

Apart from the above oncogenic lncRNAs contributing to cancer cell proliferation, invasion and metastasis, other chromatin-associated lncRNAs play a contrary role in cancer progression, and tumor suppressor lncRNAs also mainly function by regulating related gene transcription (Fig. 5B). For instance, lncRNA LINC-PINT has been identified as a tumor suppressor caRNA in many classes of cancers. Mechanistically, LINC-PINT interacts with PRC2 at a few tumor-invasion-related gene loci, such as Early Growth Response 1 (EGR1) and Integrin alpha 3 (ITGA3), and induces their silencing, ultimately repressing cancer cell migration [140]. Therefore, chromatin-associated lncRNAs play dual roles in cancer progression, which has also been confirmed for some circRNAs. For example, the oncogenic circRNA circIPO11 can recruit topoisomerase 1 (TOP1) to the GLI family zinc finger 1 (GLI1) promoter and then activate its transcription, which promotes self-renewal of liver cancer stem cells (CSCs) and promotes the progression of hepatocellular carcinoma (HCC) via Hedgehog signaling [141]. On the other hand, many tumor suppressor circRNAs participate in the inhibition of cancer cell proliferation, invasion and metastasis. Chromatin-associated circRNA circMRPS35 is demonstrated as a cancer suppressor interacting with histone modifiers in gastric cancer tissues. CircMRPS35 can specifically bind to the promoters of the FOXO1 and FOXO3a genes and be a scaffold to recruit histone acetyltransferase Lysine Acetyltransferase 7 (KAT7) depositing H4K5ac and activating FOXO1 and FOXO3a transcription, which leads to upregulation of p21 and p27 and downregulation of Twist Family BHLH Transcription Factor 1 (Twist1) and E-cadherin as well as subsequent inhibition of gastric cancer proliferation and invasion [142]. A similar intranuclear circRNA, CircGSK3B, was found to inhibit gastric cancer progression both in vitro and in vivo. CircGSK3B is able to directly interact with EZH2, suppressing the deposition of H3K27me3 on the RORA promoter, which can upregulate RORA and eventually inhibit tumor growth, invasion, and metastasis [143]. Most recent research illustrates that circNCOR1 can recruit hnRNPL to the SMAD7 promoter and directly insert into the DNA double strand to form a DNA–RNA triplex, which increases histone acetyltransferase p300-dependent H3K9ac and transcription of SMAD7, finally leading to SMAD7-mediated suppression of the TGFβ/SMAD signaling pathway and inhibition of bladder cancer (BC) cell growth as well as lymph node metastasis. Moreover, SUMOylated DExD-box helicase 39B (DDX39B)-mediated aberrant circNCOR1 export from the nucleus to the cytoplasm will decrease tumor suppression by circNCOR1 [144].

Overall, chromatin-associated ncRNAs have dual roles in tumorigenesis and cancer development and usually act as RNA scaffolds or chaperones to recruit TFs, epigenetic regulators (histone modifiers, chromatin remodelers, etc.), transcriptional coactivators, DNA topoisomerase and DNA/RNA helicase to chromatin, which is essential for modulating cancer development-associated gene transcription, downstream signal transduction and subsequent physiological processes. Recently, one of the chromatin-associated snoRNA subsets was found to be relevant to DNA damage and cancer genome instability; notably, among them, the orphan snoRNA SNORA73 can combine poly (ADP-ribose) polymerase 1 (PARP1) and the canonical H/ACA proteins DKC1/NHP2 to form a snoRNP at DNA damage genomic loci, which blocks PARP1 autoPARylation and DNA damage repair and leads to genome instability and cell differentiation in AML [145]. This suggests that chromatin-associated ncRNAs might bind to different RBPs, even DNA-binding proteins, to form RNPs on chromatin, contributing to cancer. However, some caRNAs also have a role in the cytoplasm, and their abnormal export may impair caRNA-mediated tumor suppression [144].

### CaRNAs in cancer treatments

#### CaRNAs as tumor biomarkers and therapeutic targets

In recent years, an increasing number of circulating ncRNAs, including circRNAs, lncRNAs and miRNAs, have been detected as biomarkers in the serum to predict tumor progression [146–148]. Similarly, previous findings have suggested that chromatin-associated noncoding RNAs can also be used as prognostic biomarkers. For example, LINC00525 was reported to be highly expressed in colorectal cancer, pancreatic cancer, and lung cancer and associated with higher tumor grade and poor prognosis [149, 150]. In LUAD, LINC00525 inhibits p21 gene transcription by recruiting EZH2 to the p21 promoter. In the cytoplasm, LINC00525 promotes the decay of p21 mRNA, leading to cancer cell proliferation [151]. These findings suggest that LINC00525 can be a promising therapeutic target in LUAD as well as a novel biomarker for pancancer. Additionally, a high level of LINC01271 (the human ortholog of lncRNA MaTAR25) is related to poor breast cancer patient prognosis and metastasis and can be a potential therapeutic target to inhibit tumor cell proliferation and metastasis [133]. LncRNA HOXA transcript at the distal tip (HOTTIP) was originally found to bind to WDR5 and mediate the activation of transcription by depositing H3K4me3 on the HOXA gene site [152]. Subsequent studies have found that HOTTIP is highly expressed in almost all cancers and can recruit epigenetic factors such as EZH2 or directly insert DNA double strands to form R-loops to promote cancer development [113, 153, 154]. A recent meta-analysis indicated that high expression of the caRNA HOTTIP is significantly related to poor overall survival (OS), metastasis and high tumor stage in several cancers, including osteosarcoma (OSC), gastric cancer (GC), hepatocellular carcinoma (HCC), etc. [155]. Similarly, a study demonstrated that HOTTIP is upregulated in oral squamous cell carcinoma (OSCC) patients with high tumor stages and distant metastasis [156].

On the other hand, the mechanism of caRNAs involved in tumor development may be a target for cancer therapy [157]. HOX transcript antisense RNA (HOTAIR), a classic chromatin-associated lncRNA, was first discovered to have high expression in breast cancer and to be related to metastasis and poor survival. Mechanistic research has revealed that HOTAIR can cause genome-wide retargeting of PRC2 to increase breast cancer invasiveness and metastasis [158]. To date, HOTAIR has been reported to be abnormally upregulated in at least 24 types of cancers, many of which are associated with metastasis and poor prognosis via chromatin regulation and R-loops [159]. Thus, considering the key role of HOTAIR in many cancers, it can be a promising target for diagnostics and therapeutics. Moreover, the mechanism by which some caRNAs participate in R-loop formation in cancer is becoming a potential therapeutic target. For example, the BETi JQ1 and BET degrader dBET6 can target the transcription elongation regulator BRD2 and BRD4 bromodomain, resulting in aberrant R-loop accumulation as well as DNA damage and cell death in cancer cells [114, 115]. The HDACi Romidepsin can also lead to R-loop accumulation and subsequent single-stranded DNA (ssDNA) breaks in the NCI-60 cell line, which will eventually cause DNA damage and cell death [116]. Because of the close relationship between m6A and caRNAs in cancer, targeting these caRNA-related m6A modifications and m6A readers can be a new strategy for cancer treatment. In addition, the upregulated m6A modification on ncRNAs may also serve as a diagnostic biomarker for cancer detection [119].

#### Therapeutic potential and challenges of caRNAs in cancer

Since caRNAs are widely involved in gene regulation in various cancers, compounds and nucleic acid drugs targeting caRNAs may be used for cancer treatment. Although there are currently a large number of RNA agents in phase II or III clinical development [160], no caRNA-related cancer treatments have yet entered clinical practice. Regardless of RNA drugs or compounds targeting caRNAs in cancer or the development of caRNAs as available RNA therapeutic products, there are some challenges. First, there are few reports about the tertiary structure of caRNAs having an essential role in cancer. Then, it is difficult to obtain high-resolution crystals of caRNAs binding to epigenetic factors for screening compounds or designing drugs. In addition, caRNAs may function in different ways in cancer, which also makes targeted therapy difficult. Antisense oligonucleotides (ASOs) and double-stranded small interfering RNAs (ds-siRNAs) are the most commonly used effective agents for targeting RNA [157]. Studies have shown that ASOs are more suitable for targeting lncRNAs located in the nucleus than ds-siRNAs [161], so ASOs may be more appropriate for developing nucleic acid drugs targeting caRNAs, which is also the advantage of caRNAs as therapeutic targets relative to other miRNAs, lncRNAs and circRNAs that play a role in the cytoplasm. However, RNA therapy also faces many challenges, such as the delivery efficiency of RNA reagents to specific human tumors, the immunogenicity of RNA, and the specificity of RNA binding to caRNAs [160]. Obviously, caRNAs have become promising therapeutic targets for cancer treatment, therefore, we need to find more cancer-related caRNAs and try to carry out clinical trials, and the novel insights into the relationship between chromatin-associated ncRNAs and cancer will provide more strategies for cancer treatments.

## Conclusions and perspectives

In conclusion, different types of caRNAs might have potential roles in gene regulation by directly binding to chromatin or specific proteins such as TFs, RBPs and epigenetic factors that can be connected with chromatin. In addition to caRNA-mediated transcriptional activation and repression, caRNAs especially nascent RNAs can impact genome stability and DNA damage repair in cancer progression. Various caRNAs can play a role in promoting or inhibiting cancer progression, even for the same type of caRNAs, such as chromatin-associated lncRNAs or circRNAs, which both have dual roles in cancer depending on which protein is recruited by caRNA to chromatin. Recently, an increasing number of studies have suggested that RNA m^6^A modification plays a vital role in various human cancers and could be a potential target for cancer therapy [77, 162]. Although there are few reports on the mechanism of tumorigenesis and development related to caRNAs and m^6^A, some recent studies suggest that m^6^A-deposited caRNAs or m^6^A-associated caRNAs may play an important role in tumors, which also indicates the crosstalk between RNA m^6^A modification and epigenetic mechanisms. Other RNA modifications might also be associated with caRNAs in gene regulation; for instance, pseudouridylation is the second most abundant RNA modification taking place cotranscriptionally in human pre-mRNA, and the most recent research maps the pseudouridine (Ψ) profiling of human chromatin-associated RNAs, which reveals that pseudouridine modification is abundant in alternatively spliced regions of nascent pre-mRNA and overlaps hundreds of binding sites for RBPs [163]. This indicates that pseudouridine modification on RNA might be associated with chromatin and participate in transcriptional regulation by binding RBPs. Recently, the latest technology bisulfite-induced deletion sequencing (BID-seq) has been developed to achieve quantitative sequencing of Ψ modifications in mammalian mRNA, which provides a technical foundation for studying the biological functions of Ψ modification in mRNA in many physiological and pathological processes, and more importantly, BID-seq brings RNA epitranscriptomics into a new stage [164]. With the development of new technologies and strategies to investigate RNA-chromatin interactions, we believe that different caRNA modes of action can be understood in a more comprehensive and in-depth manner and that novel mechanisms involved in gene regulation can be discovered. Further studies of caRNAs in the mechanisms of disease progression, especially cancer, will provide new insights for tumor-targeted therapy and intervention.

## Data Availability

Please contact the corresponding author for all data requests.

## Abbreviations

CaRNAs: Chromatin-associated RNAs
NcRNAs: Non-coding RNAs
MARGI: Mapping RNA-Genome Interactions
ChAR-seq: Chromatin-associated RNA Sequencing
RADICL-seq: RNA And DNA Interacting Complexes Ligated and sequenced
GRID-seq: Global RNA interactions with DNA by deep sequencing
LncRNAs: Long non-coding RNAs
CircRNAs: Circular RNAs
SnRNAs: Small nuclear RNAs
SnoRNAs: Small nucleolar RNAs
ERNAs: Enhancer RNAs
PaRNAs: Promoter-associated RNAs
AsRNAs: Antisense RNAs
TFs: Transcription Factors
RBPs: RNA-binding proteins
PRC2: Polycomb Repressive Complexes 2
IPSCs: Induced pluripotent stem cells
G4: G-quadruplex
DDX21: DExD-Box Helicase 21
TSS: Transcription start site
MiRNA: MicroRNA
TERRA: Telomeric-repeat-containing RNA
TCF21: Transcription Factor 21
GADD45A: Growth Arrest And DNA Damage Inducible Alpha
TET1: Tet methylcytosine dioxygenase 1
RNPs: Ribonucleoproteins
YY1: Yin Yang 1
IDH1: Isocitrate dehydrogenase 1
MuSCs: Muscle stem cells
HDAC3: Histone deacetylase 3
hnRNP K: Heterogeneous nuclear ribonucleoprotein K
EIciRNAs: Exon-intron circRNAs
BRD4: Bromodomain Containing 4
ZCCHC4: Zinc finger CCHC-type-containing 4
ELAVL1/HuR: ELAV Like RNA Binding Protein 1
ALKBH5: AlkB Homolog 5
FTO: Fat mass and obesity-associated protein
m6A: N6-methyladenosine
carRNAs: Chromatin-associated regulatory RNAs
NEXT: Nuclear exosome targeting
LINE1: Long interspersed nuclear elements 1
TRIM28/KAP1: Tripartite Motif Containing 28/KRAB-associated protein
SETDB1: SET Domain Bifurcated Histone Lysine Methyltransferase 1
KDM3B: Histone lysine demethylase 3B
RBM15: RNA-binding motif protein 15
TRCs: Transcription-replication conflicts
BRCA1/2: Breast cancer type 1/2 susceptibility protein
DSBs: DNA double-strand breaks
RNF168: Ring Finger Protein 168
DHX9: DExH-Box Helicase 9
PARPi: PARP inhibitors
TopBP1: Topoisomerase II Binding Protein 1
EWSR1: EWS RNA binding protein 1
FLI1: Friend leukaemia integration 1 transcription factor
ADAR1: Adenosine deaminase acting on RNA 1
DDX41: Dead box helicase 41
AML: Acute myeloid leukemia
BC: Bladder cancer
CBSs: CTCF-binding sites
TAD: Topologically associated domain
BETis: BET inhibitors
HDACis: Histone deacetylases inhibitors
EIF3C: Eukaryotic Translation Initiation Factor 3 Subunit C
SPRED2: Sprouty Related EVH1 Domain Containing 2
STAT3: Signal Transducer And Activator Of Transcription 3
GSCs: Glioblastoma stem-like cells
FOXM1: Forkhead Box M1
ARHGAP5: Rho GTPase Activating Protein 5
GATA3: GATA Binding Protein 3
LCAT3: Lung Cancer Associated Transcript 3
FUBP1: Far Upstream Element Binding Protein 1
FXR1: FMR1 Autosomal Homolog 1
OXPHOS: Oxidative phosphorylation
RGS1: Regulator of G Protein Signaling 1
CCL17: Thymus and activation-regulated chemokine
TARC: PAK2, P21 (RAC1) Activated Kinase 2
KCNMA1: Potassium Calcium-Activated Channel Subfamily M Alpha 1
PURB: Purine rich element binding protein B
E2F1: E2F Transcription Factor 1;
NRF1: Nuclear Respiratory Factor 1
CRC: Colorectal cancer
ALCL: Anaplastic large cell lymphoma
LSH: Lymphoid-specific helicase
EZH2: Enhancer of zeste homolog 2
CDKN1B: Cyclin Dependent Kinase Inhibitor 1B
EGR1: Early Growth Response 1
ITGA3: Integrin alpha 3
TOP1: Topoisomerase 1
GLI1: GLI Family Zinc Finger 1
CSCs: Cancer stem cells
HCC: Hepatocellular carcinoma
KAT7: Lysine Acetyltransferase 7
Twist1: Twist Family BHLH Transcription Factor 1
PARP1: Poly;ADP-ribose polymerase 1
GC: Gastric cancer
OS: Overall Survival
ASOs: Antisense oligonucleotides
Ds-siRNAs: Double-stranded small interfering RNAs
